# Discrimination of grass pollen of different species by FTIR spectroscopy of individual pollen grains

**DOI:** 10.1007/s00216-020-02628-2

**Published:** 2020-04-29

**Authors:** Sabrina Diehn, Boris Zimmermann, Valeria Tafintseva, Murat Bağcıoğlu, Achim Kohler, Mikael Ohlson, Siri Fjellheim, Janina Kneipp

**Affiliations:** 1 Department of Chemistry, Humboldt-Universität zu Berlin, Brook-Taylor-Straße 2, 12489 Berlin, Germany; 2 Faculty of Science and Technology, Norwegian University of Life Sciences, 1432 Ås, Norway; 3 Faculty of Environmental Sciences and Natural Resource Management, Norwegian University of Life Sciences, 1432 Ås, Norway; 4 Faculty of Biosciences, Norwegian University of Life Sciences, 1432 Ås, Norway

**Keywords:** Poaceae, Pollen, Fourier-transform infrared (FTIR) microspectroscopy, Mie scattering, Paraffin, Non-negative matrix factorization, Extended multiplicative signal correction, Partial least squares-discriminant analysis, Machine learning

## Abstract

Fourier-transform infrared (FTIR) spectroscopy enables the chemical characterization and identification of pollen samples, leading to a wide range of applications, such as paleoecology and allergology. This is of particular interest in the identification of grass (Poaceae) species since they have pollen grains of very similar morphology. Unfortunately, the correct identification of FTIR microspectroscopy spectra of single pollen grains is hindered by strong spectral contributions from Mie scattering. Embedding of pollen samples in paraffin helps to retrieve infrared spectra without scattering artifacts. In this study, pollen samples from 10 different populations of five grass species (*Anthoxanthum odoratum*, *Bromus inermis*, *Hordeum bulbosum*, *Lolium perenne*, and *Poa alpina*) were embedded in paraffin, and their single grain spectra were obtained by FTIR microspectroscopy. Spectra were subjected to different preprocessing in order to suppress paraffin influence on spectral classification. It is shown that decomposition by non-negative matrix factorization (NMF) and extended multiplicative signal correction (EMSC) that utilizes a paraffin constituent spectrum, respectively, leads to good success rates for the classification of spectra with respect to species by a partial least square discriminant analysis (PLS-DA) model in full cross-validation for several species. PLS-DA, artificial neural network, and random forest classifiers were applied on the EMSC-corrected spectra using an independent validation to assign spectra from unknown populations to the species. Variation within and between species, together with the differences in classification results, is in agreement with the systematics within the Poaceae family. The results illustrate the great potential of FTIR microspectroscopy for automated classification and identification of grass pollen, possibly together with other, complementary methods for single pollen chemical characterization.

**Electronic supplementary material:**

The online version of this article (10.1007/s00216-020-02628-2) contains supplementary material, which is available to authorized users.

## Introduction

Many research fields, including paleobiology, climate research, and allergology, rely on a fast and reliable identification of pollen [1–4]. Furthermore, insight into pollen chemical composition is important for any plant-related phenotyping, crucial in agriculture, plant physiology, and ecology, e.g., when adaptation of plants to altered environmental conditions is discussed [5, 6]. Traditional pollen analysis is done by light or electron microscopy and is based on the morphology of pollen grains, specifically their shape and size, position, and shape of apertures (pores), as well as texture and morphologyof the cell wall [7]. In most cases, identification to species level is not possible, and some pollen types can be identified only to higher taxonomic level, such as family level for grasses (Poaceae) [8]. For that reason, spectroscopic and spectrometric methods, including mass spectrometry, Raman scattering, and Fourier-transform infrared (FTIR) spectroscopy, are currently being harnessed by several groups in order to obtain not only more precise identification but also high-throughput pollen chemical analysis [9–13].

As demonstrated during the last decade, FTIR spectroscopy enables a detailed analysis of the species-specific chemical composition of pollen [14–21]. FTIR analysis of pollen is based on the fingerprint-like characteristics of the IR spectrum, containing contributions from all different kinds of biomolecular constituents. More recently, FTIR spectroscopy was shown to allow for a characterization of chemical variation also at the subspecies level, specifically between populations of the same pollen species, and led to conclusions regarding, e.g., the adaptation of plant populations to environmental conditions [22–28]. Although the majority of FTIR pollen studies were conducted by measurement of bulk pollen samples, containing 1 mg or more of pollen sample per measurement, some studies have used FTIR microspectrometers as well [5, 15, 18, 20, 28–33]. FTIR microspectroscopy measurements of complex mixtures of pollen grains of different plant species or various particulate impurities are also possible. Unfortunately, FTIR microspectroscopy of single pollen grains provides specific challenges, as scattering effects occur for the mid-IR wavelengths due to the micron-scale size of typical pollen grains [15, 20, 29, 31]. The spectral contribution from Mie scattering, as well as other artifacts, can superimpose the absorbance spectrum, depending on the geometry of the sample, and cause band shifts, distortions, and artificial bands [34]. These scattering problems can be addressed by numerical analytical approaches, such as model-based spectral preprocessing [30] and spectral averaging [32], or by modifying experimental settings, such as measurements of many pollen grains with large microscope apertures [15, 18, 20] or measurement in an embedding matrix [31].

Extended multiplicative signal correction (EMSC) is a model-based spectral preprocessing method [35] that can take scattering contributions into account and separates them from the molecular absorption [35]. When applied to FTIR spectra, EMSC retrieves chemical information [36, 37]. However, due to the heterogeneity of pollen shapes, sizes, chemistry, and surface texture, a successful modeling of the physical contributions on single pollen grain FTIR spectra is challenging, even by novel EMSC-based algorithms [30].

Recently, a strategy to obtain FTIR microspectra of single pollen grains using paraffin embedding was presented [31]. Embedding in soft paraffin leads to a suppression of the scattering effects due to the similar refractive index of paraffin and the pollen grains [31]. A successful discrimination between pollen with a very broad phylogenetic background, including one grass species, was obtained [31]. This approach is aligned with the traditional pollen sampling and measurement, since soft paraffin is used in standard aero-biology and aero-allergology pollen traps. In that study, the strong spectral contribution of paraffin was resolved by cutting out the region in the spectra that had strong paraffin signals [31]. Although pollen spectral classification was successful, retrieval of pollen signals in the removed spectral region and suppression of less prominent paraffin contributions in other spectral regions would be very useful. Managing the presence of paraffin in biosamples analyzed by FTIR microspectroscopy has been an ongoing discussion, in particular in the context of tissue diagnostics, since paraffin embedding is a routine procedure in histopathology as well [38, 39]. Strategies include the mathematical removal of the paraffin signals, e.g., by EMSC [40], by independent component analysis [41], or by partial least squares [42].

Here, we discuss the possibilities to utilize FTIR microspectra of paraffin-embedded single grass pollen grains to distinguish between pollen from five grass species within the Pooideae subfamily of the Poaceae family. Pooideae comprise some of the economically most important plant species such as wheat, rye, and barley. Pooideae also have harmful impact, their pollen being one of the most widespread causes of hay fever, allergic rhinitis, and asthma [43]. In the work presented here, we have measured ~ 1000 spectra of pollen of *Anthoxanthum odoratum, Bromus inermis, Hordeum bulbosum, Lolium perenne*, and *Poa alpina*, with each of these species being represented by 10 individual plants from two populations. This is an extremely challenging sample set for traditional pollen identification and classification, since the studied species have almost identical pollen morphology. A recent study on pollen samples of eight grass species in measurements with large aperture, covering 8–10 grains has demonstrated the great potential of FTIR microspectroscopy for identification of grass pollen [20]. Here, we now push the FTIR microspectroscopy approach closer to real world paleoecological and allergological samples, by classifying individual pollen grains of grasses.

**Scheme 1:**
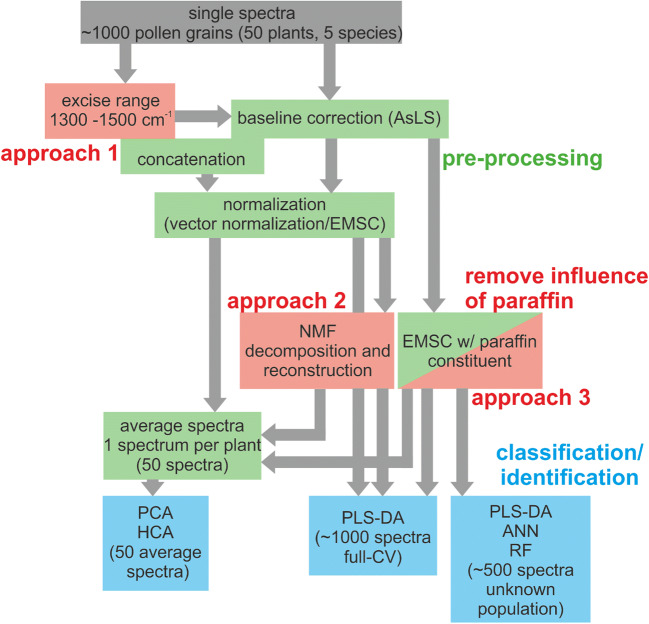
Schematic representation of the data analysis conducted with ~ 1000 FTIR microspectra of single pollen grains from five Poaceae species. Basic preprocessing steps are marked in green. Preprocessing steps that concern suppression of spectral contributions of the paraffin are marked in red color. Classification analyses are marked in blue. The preprocessing steps depend on the approach that is used for paraffin correction, and on the classification analysis, as indicated by the arrows. Abbreviations: ANN, artificial neural network; EMSC, extended multiplicative signal correction; HCA, hierarchical cluster analysis; NMF, non-negative matrix factorization; PCA, principal component analysis; PLS-DA, partial least square discriminant analysis; RF, random forest

First, a comparative study comprising different spectral preprocessing approaches was conducted in order to assess and suppress spectral contributions of the paraffin-embedding matrix in classification analyses (Scheme 1). Apart from simple baseline correction and normalization that does not alter the influence of the paraffin contributions, the preprocessing approaches included (1) omitting of the spectral region containing the most dominant paraffin signals, as proposed previously [31]; (2) separation of the contribution by paraffin from that of the pollen constituents by non-negative matrix factorization (NMF); and (3) an EMSC approach with modeling of the paraffin spectral contributions as suggested in a previous work [44] (Scheme 1, red colored boxes).

Second, characterization of the spectral differences between the pollen of the different species was done by principal component analysis (PCA) and hierarchical cluster analysis (HCA). Lastly, a classification analysis with three machine learning classifiers, namely partial least square discriminant analysis (PLS-DA), artificial neural network (ANN), and random forest (RF), was conducted on a large independent sample set. The analysis of the spectral variance within and between the species, together with a comparison of success rates in PLS-DA, ANN, and RF, underpins the sensitivity of FTIR microspectroscopy to characterize compositional differences between grass species, and to relate them to systematics/phylogenetic information at the level of individual pollen grains.

## Materials and methods

### Pollen samples

The sample set of this study contains pollen from two populations from each of the five Poaceae species *Anthoxanthum odoratum* (accessions 51541 and 63063 from Millennium Seed Bank), *Bromus inermis* (accessions NGB2875 and NGB5420 from the Nordic Gene Bank), *Hordeum bulbosum* (accessions PI614642 and PI639320 from Germplasm Resources Information Network (GRIN), United States Department of Agriculture), *Lolium perenne* (accessions NGB4262 and NGB14263 from the Nordic Gene Bank), and *Poa alpina* (accessions NGB1197 and NG6297 from the Nordic Gene Bank)*.* From each population, up to five individuals of different genotypes were used in the experiment.

Seeds from *Anthoxanthum odoratum* and *Poa alpina* were germinated in moist soil (Tjerbo Gartnerjord, Tjerbo, Rakkestad, Norway) in an open greenhouse in the spring. The plants grew over summer and were subsequently vernalized for 12 weeks at 4 °C with a day length of 8 h. Following vernalization, the day length was increased to 16 h to induce flowering. The plants were grown at 20 °C until flowering. During this period, the plants were fertilized twice a week with water containing 4% Yara Kristalon Indigo (Yara, Skøyen, Norway) and 3% YaraLiva calcium nitrate (Yara, Skøyen, Norway) adjusted to an electron conductivity of 1.5. For *Bromus inermis*, *Hordeum bulbosum*, and *Lolium perenne*, seeds were stratified in moist soil (Tjerbo Gartnerjord, Tjerbo, Rakkestad, Norway) in the dark for 6 days, first at 4 °C for 5 days, followed by 1 day at room temperature. Seeds were then transferred to an open greenhouse in long days (16 h day length) at 17 °C and grown for 4 weeks, before temperature (vernalization at 4 °C for 6 weeks and then transferred to 17 °C, or no vernalization at 17 °C) and day lengths (8 or 16 h photoperiod) were varied for different plants as required by another study from which the plants were sampled. The plants were fertilized regularly during the course of the experiment.

Pollen were collected from the plants at the onset of pollination (varying for each species and growth condition) and stored at − 20 °C. The complete set of plants contains in total 50 individuals, with 10 individual plants for each species. Approximately 20 different pollen grains were sampled from each plant.

### Sample preparation and data acquisition

For FTIR microspectroscopy, the pollen grains were spread onto a thin layer of paraffin on a ZnSe slide. With the help of a glass slide, the soft paraffin (Enzborn Vaseline, Nordwalde, Germany) was distributed over the pollen grains, resulting in embedding of the pollen grains in the thin paraffin layer. FTIR spectra were obtained in transmission mode using a Nicolet FTIR microscope (Thermo Scientific, Waltham, USA), equipped with a single element MCT detector and with a × 32 Cassegrainian objective. The size of the sampled spot was 15 μm × 15 μm. As light source, a synchrotron source (beam line IRIS, HZB-BESSY, Berlin) was used. The FTIR spectra were measured with a spectral resolution of 4 cm^−1^ and digital spacing of 1.9 cm^−1^, by averaging 128 interferograms per spectrum. A background spectrum was collected from the ZnSe slide with identical parameters. From each of the 50 plants, approximately 20 different pollen grains were measured (with one spectrum per pollen grain), resulting in a data set of 1004 spectra in total. Moreover, for each plant sample, 2 to 5 spectra of the pollen-free paraffin layer were measured using the same condition as described above, leading to 190 pure paraffin spectra. Finally, individual pollen grains were measured on a ZnSe slide without paraffin embedding (i.e., unembedded samples). Approximately 20 spectra of individual pollen grains from only one plant per grass species were measured, resulting in 97 spectra of unembedded samples in total.

### Spectral preprocessing

Scheme 1 outlines the data processing steps that include basic preprocessing such as baseline correction, normalization, and calculation of average spectra (Scheme 1, green boxes), as well as the steps that were used specifically to assess the contributions by paraffin to the spectra (Scheme 1, red boxes). For the analysis of the spectral sets, the spectral region of 1800 to 800 cm^−1^ was selected, since it contains bands that are distinctive for pollen grains [15, 16, 22]. Three spectral preprocessing approaches were tested to assess and suppress paraffin spectral contributions in the spectral set of paraffin-embedded pollen grains (Scheme 1, red colored boxes). Different preprocessing was applied on the spectral set of paraffin-embedded samples, depending on the specific approach for paraffin correction (Scheme 1, different arrows).

#### Preprocessing before comparison of spectra from paraffin-embedded and non-embedded samples

In order to compare the spectra from non-embedded and paraffin-embedded samples, all spectral sets belonging to non-embedded and paraffin-embedded pollen grains were preprocessed as follows: The spectra were baseline-corrected using asymmetric least squares (AsLS) correction, as proposed by Eilers [45] and vector-normalized before averaging.

#### Preprocessing for an analysis without observing the influence of the paraffin contribution

For simple baseline correction and normalization, the spectra from paraffin-embedded pollen grains are baseline-corrected by AsLS before applying a simple EMSC, an MSC model extended by a linear and quadratic component [46], that replaces normalization. Afterwards, the spectra were smoothed using a Savitzky–Golay filter with a window size of 9 and a second-order polynomial. The optimization of the Savitzky–Golay parameters was accomplished as described in [46], using PLS-DA of the spectra from two pollen species permutatively, which resulted in a median window size of 9. For classification by PLS-DA, the individual spectra were used. For analysis by HCA and PCA, averages of the spectra of one respective plant were calculated.

#### Preprocessing in application of approach 1 (cf. Scheme 1)

The spectral region 1300–1500 cm^−1^ was omitted from the spectra of the embedded pollen grains, thus dividing the data into the two ranges 800–1300 and 1500–1800 cm^−1^. Before concatenation of the two ranges, each range was baseline-corrected using AsLS correction. After concatenation, EMSC was applied as described above, also leading to normalization, and Savitzky–Golay smoothing as applied. For classification by PLS-DA, the individual spectra were used. For analysis by HCA and PCA, averages of the spectra of one respective plant were calculated.

#### Preprocessing in application of approach 2 (cf. Scheme 1)

The spectra of the embedded pollen grains were baseline-corrected using AsLS correction. After subsequent vector normalization, NMF was used to split each spectrum into a paraffin and a pollen component in order to eliminate the paraffin spectral signature. The 1004 pollen spectra and the 190 pure paraffin spectra were decomposed together into six components using the *nnmf* function in Matlab. All components that contained paraffin signals on visual inspection were separated from those without prominent paraffin signature and left out in the reconstruction of 1004 spectra without paraffin contribution. For classification by PLS-DA, the individual spectra were used. For analysis by HCA and PCA, averages of the spectra of one respective plant were calculated.

#### Preprocessing in application of approach 3 (cf. Scheme 1)

The AsLS baseline-corrected spectra of the embedded pollen grains were corrected by the complex EMSC model using a linear and a quadratic component, extended by a representative spectrum of paraffin, as suggested by Kohler et al. [44] (Scheme 1, red-green box). In contrast to the simple EMSC model used in the preprocessing of the spectra treated by *approach 1* and by the untreated spectra (Scheme 1), where an average spectrum is used in the model, in the complex EMSC model, we assume two different constituents in the spectra, specifically the paraffin constituent and the pollen constituent. For the representative spectrum of paraffin for the EMSC model, an average spectrum was calculated from the 190 pure paraffin spectra. For classification by PLS-DA, the individual spectra were used. For analysis by HCA and PCA, averages of the spectra of one respective plant were calculated.

All spectral preprocessing was performed using Matlab (MathWorks, Inc.).

### Unsupervised data analyses

Averages of all spectra (pollen grains) from an individual plant, resulting in 10 average spectra per species, were, after correction for paraffin signals by the different approaches, and also without correction for paraffin signals, analyzed using HCA and PCA (Scheme 1, left blue box). The full spectral range from 800 to 1800 cm^−1^ was used for the analyses. HCA was executed using Euclidean distances and Ward’s algorithm.

All unsupervised data analyses were obtained using Matlab (MathWorks, Inc.).

### Classification data analyses

In order to assess the three approaches for elimination of the influence of paraffin signals, the data sets comprising 1004 (1003, in the case of approach 1) preprocessed pollen spectra were analyzed using PLS-DA with an optimized amount of latent variables, using 10-fold cross-validation. We trained each model using the whole data set except one spectrum and permutated this procedure to apply leave-one-out cross-validation (full CV) (Scheme 1, middle blue box).

The classification analyses were conducted by splitting the spectral data set in half, where each comprised the spectrum of only one population per species, thus creating a fully independent training and test sets with 502 spectra each (Scheme 1, right blue box). The preprocessed data set, obtained by approach 3, was selected as optimal for the classification analyses based on the aforementioned PLS-DA with full CV. Three different machine learning classifiers were used in the analyses: PLS-DA, ANN, and RF.

A feed-forward ANN containing 519 input neurons, 50 neurons in the hidden layer, and 5 outputs corresponding to the species was constructed and trained using the *patternnet* and *train* functions in Matlab. Of the 502 training spectra, 70% were used for training, 25% for validation, and 5% for internal testing. Success rates for ANN identification were calculated for a set of 502 spectra comprising the data from the other respective population of each species. RF classification was applied by using the *treebagger* function in Matlab with 300 trees on the 502 training spectra of one population of each species. The classification of the 502 spectra from the test set was executed using the *predict* function.

All classification analyses were obtained using the Statistics and Machine Learning Toolbox, as well as the Neural Network Toolbox in Matlab (MathWorks, Inc.).

## Results and discussion

### Pollen morphology

**Fig. 1:**
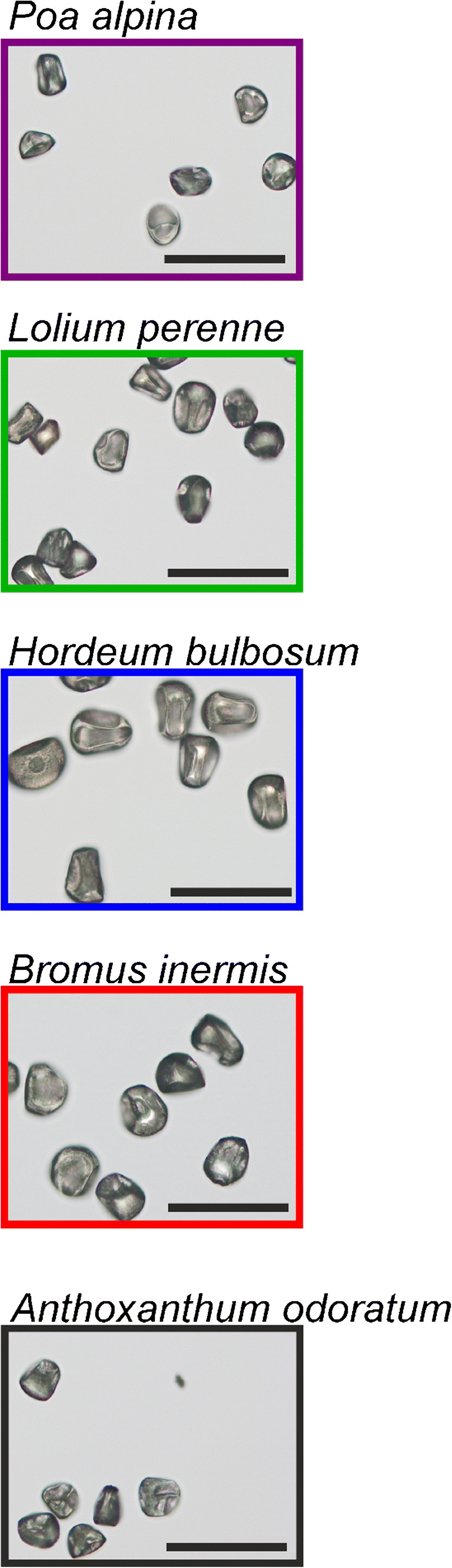
Bright-field micrographs (× 20) of pollen grains from the five indicated grass species used. Scale bars 100 μm

As can be seen in the bright-field images (Fig. 1), the dry pollen grains from the five different grass species are similar in size and morphology. In general, grass pollen is characterized by a simple spherical shape, single circular and annulate aperture situated distally, and microechinate grain wall ornamentation [8]. Grass pollen has very limited mechanisms for preventing desiccation [47]. As a result, grass pollen morphology is dramatically changed after shedding, collapsing from a spherical shape of fresh pollen to extensive infolding of dry pollen [48]. The extensive infolding leads to large variation in Mie scattering effects, resulting with extremely unreproducible spectra. Although the pollen grains of all five measured species have similar morphology, those of *Poa alpina* and *Anthoxanthum odoratum* are slightly smaller than the pollen grains of *Lolium perenne*, *Bromus inermis*, and *Hordeum bulbosum*.

### Influence of the paraffin spectral contribution

**Fig. 2:**
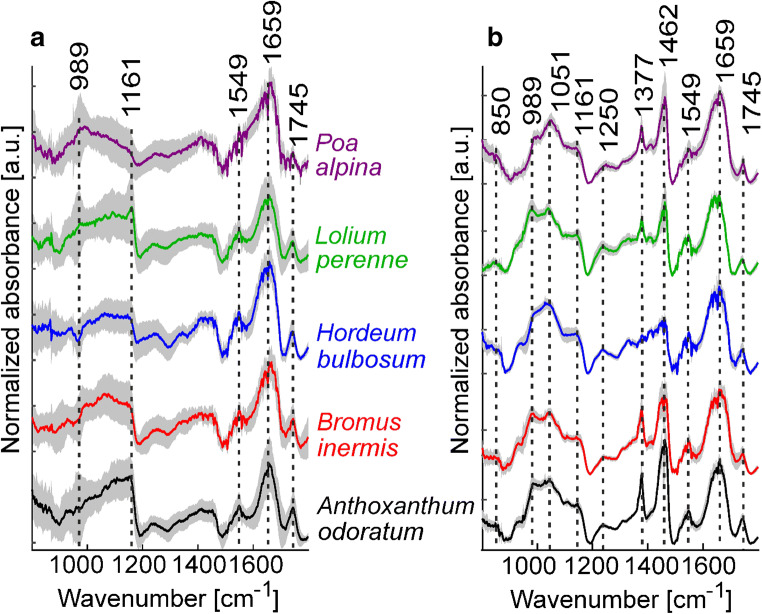
Average FTIR spectra of five grass species (based on 20 individual pollen grains per plant, and 10 plants per species): **a** unembedded samples and **b** paraffin-embedded samples. The standard deviation for each group of spectra is indicated in gray

Following our recently established protocol [31], we embedded the pollen samples in paraffin to avoid scattering artifacts in the spectra. Figure 2 shows the averages of baseline-corrected and vector-normalized spectra of non-embedded (Fig. 2a) and of the paraffin-embedded pollen grains (Fig. 2b) for each pollen species. The spectra of the embedded pollen show much less variation within each species (Fig. 2b) compared to the large standard deviation when measured as the unembedded samples (Fig. 2a). The most prominent bands in the spectra are found at 989 and 1045 cm^−1^ both assigned to carbohydrates, at 1161 cm^−1^ assigned to lipids and carbohydrates, at 1549 and 1659 cm^−1^ assigned to amide II and amide I vibrations of proteins, respectively, and at 1745 cm^−1^ assigned to lipids [18]. In Fig. 2 b, the characteristic absorbance of paraffin adds to this pollen signature and is particularly prominent in the region from 1300 to 1500 cm^−1^. In particular, bands associated with the methyl rocking vibration at 1377 cm^−1^ and the CH_2_ bending and CH_3_ deformations modes at 1462 cm^−1^ determine the spectra of all the embedded pollen samples (Fig. 2b) [49]. Although much less dominating, the spectra from the non-embedded samples also contain signals in this spectral region.

The paraffin bands at 1377 and 1462 cm^−1^ in the spectra of the embedded samples vary between the different species (Fig. 2b). In the spectra of pollen from *Poa alpina* and *Anthoxanthum odoratum*, both bands have higher relative absorbance values, whereas for *Lolium perenne* and *Bromus inermis*, they are less strong. In the spectrum of *Hordeum bulbosum*, the two characteristic paraffin signals have much smaller contributions and the spectrum in the region from 1300 to 1500 cm^−1^ resembles that of the averaged spectrum from the non-embedded pollen grains (compare the two blue traces in Fig. 2 a and b). The different relative contribution by the embedding paraffin in the spectra of the different species is likely related to the different size of the pollen grains, leading to a different relative amount of paraffin versus pollen material in the probed microscopic volume.

We have tested three different approaches for correction of FTIR spectra of the paraffin-embedded samples. In comparison, in the simplest procedure, paraffin spectral contributions were not suppressed, and the spectra were only baseline-corrected and vector-normalized. The assessment of this preprocessing by PLS-DA with full CV indicates clearly that the spectra of the different species can be discriminated (Table 1). The overall success rate of 79% was achieved, with the individual success rates of approximately 90% for *Hordeum bulbosum*, *Anthoxanthum odoratum*, and *Poa alpina* spectra. The average spectra in Fig. 2 already suggest that the different extent of the paraffin spectral contribution could also influence the discrimination of the different pollen species. The results of the PCA corroborate this, and the loadings of the first principal component (PC 1) (see Electronic Supplementary Material (ESM) Fig. S1, right column) show the two strong paraffin-related signals at 1377 and 1462 cm^−1^. Also, in the other principal components, e.g., PC 4 (ESM Fig. S1, right column), the paraffin signals may be a reason for the species-related variation, as can be seen from the presence of signal at 1460 cm^−1^. This indicates that the paraffin contribution needs to be minimized before data analysis, in order to obtain classification and identification based on pollen chemistry alone.

**Table 1:** Result of PLS-DA classification of spectra from paraffin-embedded pollen after simple baseline correction and vector normalization. Nine latent variables were used. The results are based on full cross-validation

Output class	Target class				
	*A. odoratum*	*B. inermis*	*H. bulbosum*	*L. perenne*	*P. alpina*
*A. odoratum*	184	3	5	13	11
*B. inermis*	1	114	10	14	2
*H. bulbosum*	4	51	189	5	2
*L. perenne*	5	18	0	131	7
*P. alpina*	5	14	5	33	178
Success rate (SR)	92%	57%	90%	67%	89%
Overall SR	79%				

### Selection of non-affected spectral ranges

As discussed above, the strong deformation modes of paraffin affect the spectra mostly in the spectral range from 1300 to 1500 cm^−1^ with the two bands at 1377 and 1462 cm^−1^. Therefore, this spectral region was omitted from the data set (compare Scheme 1, approach 1), similar to the approach in our first paraffin-embedding study [16]. Eliminating only the two strongest bands from paraffin here, we assume that other spectral features contributed by the paraffin in the regions 800–1300 and 1500–1800 cm^−1^ are negligibly small compared to the absorption bands of the pollen samples themselves. Following the removal of the 1300–1500-cm^−1^ spectral range, the spectra were normalized by the simple EMSC model.

The assessment by PLS-DA with full CV shows that the overall classification success rate is lower (i.e., 76%, see Table 2) compared to the preprocessing, where the contribution by paraffin is not corrected for (Table 1). Similar to these results, the success rates can vary enormously for each of the pollen species, ranging from 46% for *Bromus inermis*, where one fourth of the actual *Bromus inermis* pollen spectra was misclassified as *Hordeum bulbosum*, to 91% correct classification of *Anthoxanthum odoratum* and *Poa alpina* spectra. PCA results show that the main variances within this data set are found in the spectral range from 850 to 1150 cm^−1^ (ESM Fig. S2A right loadings of PC 1 and PC 2), which can be assigned mainly to carbohydrates [14, 18]. A differentiation between the pollen spectra from *Anthoxanthum odoratum* and *Poa alpina* and between *Hordeum bulbosum* and *Lolium perenne* can be achieved in PC 3 and PC 6, respectively, as found in the scores plot (ESM Fig. S2B). The finding that the spectral differences in the pollen spectra preprocessed by excluding the range from 1300 to 1500 cm^−1^ lead to a relatively small drop in classification success rates, compared to the simple preprocessing—without consideration of the paraffin influence, is in agreement with a previous work that reports the successful discrimination of paraffin-embedded pollen from other plant species [31].

**Table 2:** Result of PLS-DA classification of spectra from paraffin-embedded pollen corrected by omitting the spectral range from 1300 to 1500 cm^−1^, following approach 1 (cf. Scheme 1). Nine latent variables were used. The results are based on full cross-validation

Output class	Target class				
	*A. odoratum*	*B. inermis*	*H. bulbosum*	*L. perenne*	*P. alpina*
*A. odoratum*	181	9	6	16	8
*B. inermis*	6	91	13	14	6
*H. bulbosum*	4	52	183	14	0
*L. perenne*	4	28	1	126	4
*P. alpina*	4	19	6	26	182
Success rate (SR)	91%	46%	88%	64%	91%
Overall SR	76%				

### Decomposition of spectra from paraffin-embedded pollen using NMF

**Fig. 3:**
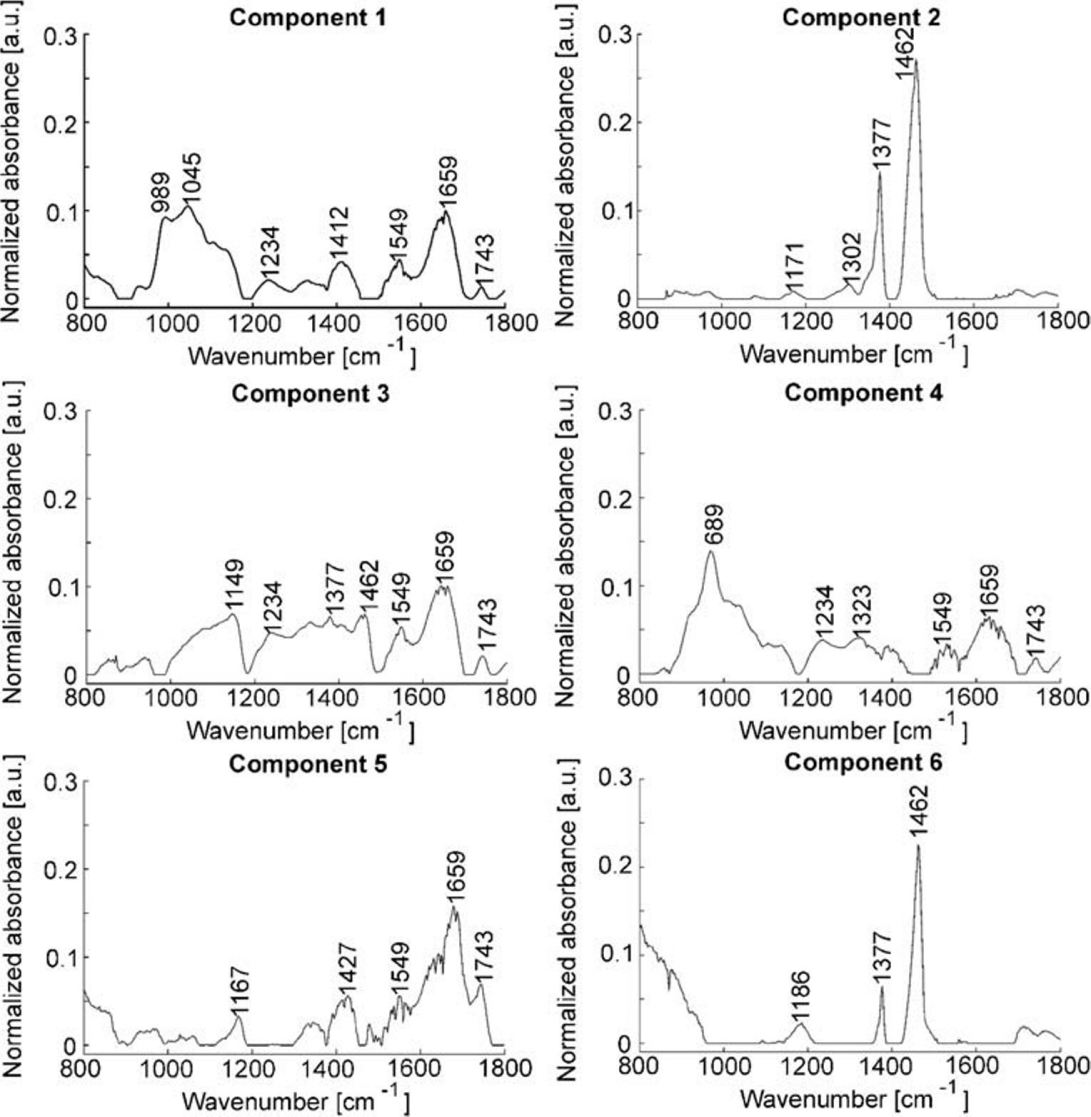
First six components of the spectral decomposition by non-negative matrix factorization (based on 1004 spectra of paraffin-embedded pollen grains, and 190 pure paraffin spectra, compare Scheme 1, approach 2). Components 2 and 6 show typical contributions by paraffin. See Table 3 for the relative contribution of the six components in the different pollen species

**Fig. 4:**
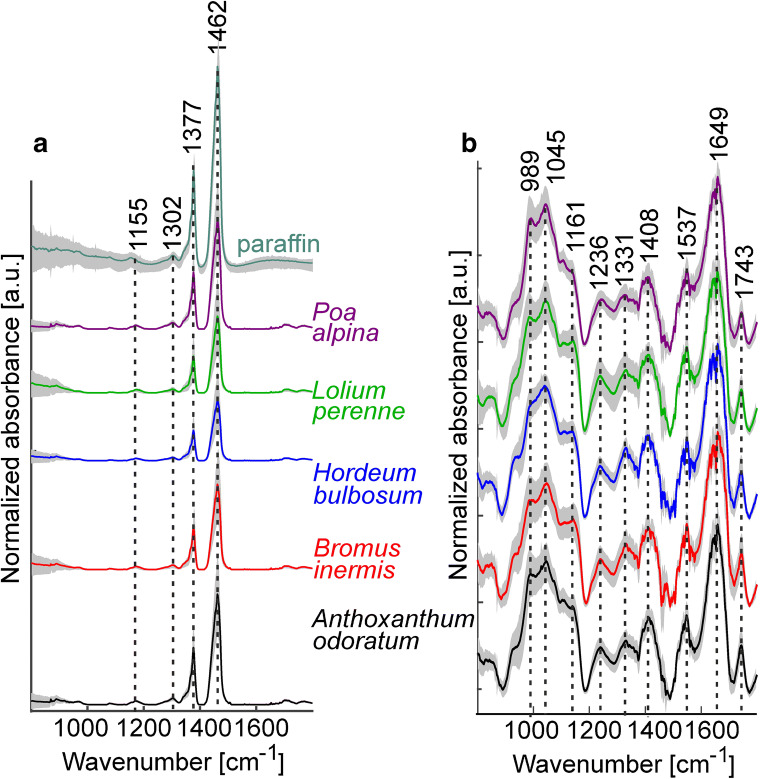
**a** Spectra obtained by reconstruction from component 2 and component 6 upon non-negative matrix factorization (NMF) with six components (cf. Fig. 3) for each species, revealing the typical paraffin signature. An average of 190 pure paraffin spectra is shown for comparison (top). **b** Reconstructed spectra from NMF components 1, 3, 4, and 5 for each species. All spectra are averages of 200 individual reconstructed spectra (corresponding to 200 pollen grains). The standard deviation for each group of spectra is marked in gray. See Fig. 3 and Table 3 for details on NMF components

A decomposition of spectral signatures belonging to different chemical constituents within the same spectrum of a complex sample can be achieved by a matrix factorization algorithm, such as NMF. This can result in a more detailed analysis of the spectral features from the different chemical constituents [50]. In addition, such matrix factorization algorithms have been shown to be very useful for the exclusion of disruptive contributions from spectra, e.g., for de-noising [51, 52]. Therefore, NMF was used in another preprocessing approach (Scheme 1, approach 2) to split our spectra into pollen spectra and paraffin spectra. In this procedure, the 1004 spectra from each individual pollen grain and 190 spectra of pure paraffin were decomposed together several times using different numbers of components—six components. The decomposition using six components was chosen as optimal based on visual inspection, which indicated a good separation of the paraffin spectra in components 2 and 6 (Fig. 3). These two components show the typical paraffin bands at 1377 and 1462 cm^−1^. The reconstructed paraffin and pollen spectra were obtained for each measured spectrum (each pollen grain), and the averages of these two sets of reconstructed spectra for each species are shown in Fig. 4 a and b, respectively. The reconstructed paraffin spectra (Fig. 4a) are in good agreement with a paraffin reference spectrum (Fig. 4a, top). Table 3 shows the normalized relative amount of each of the six components. The variation of the relative paraffin contribution (Table 3, components 2 and 6) is in good agreement with the visual observation of pollen spectra (Fig. 2), showing its larger contribution to *Anthoxanthum odoratum* and *Poa alpina* spectra and smaller contribution for the other three species. The averages of the spectra that were reconstructed from the remaining four components show no characteristic paraffin signals (Fig. 4b). Compared to the spectra from unembedded single pollen grains on ZnSe slide discussed above (compare Fig. 2a), three characteristic bands at 1236, 1331, and 1408 cm^−1^ are visible more clearly. They can be assigned to phospholipids, indicated, e.g., by the P=O-stretching vibration at 1236 cm^−1^, amino acids, as illustrated by the COO^−^ stretching mode at 1408 cm^−1^, and carbohydrates, the latter possibly causing the band at 1331 cm^−1^ that is likely assigned to a ring deformation vibration [18, 49, 53].

**Table 3:** Averaged relative spectral contribution of each component after decomposition using NMF (cf. Scheme 1, approach 2). The spectral contribution is averaged for each pollen species

	Comp 1 [%]	Comp 2 [%] (Paraffin)	Comp 3 [%]	Comp 4 [%]	Comp 5 [%]	Comp 6 [%] (Paraffin)
*A. odoratum*	41 ± 12	25 ± 9	12 ± 9	12 ± 11	8 ± 6	2 ± 4
*B. inermis*	34 ± 13	18 ± 9	15 ± 12	13 ± 9	17 ± 11	3 ± 5
*H. bulbosum*	36 ± 11	13 ± 7	19 ± 9	17 ± 8	12 ± 8	2 ± 3
*L. perenne*	34 ± 16	15 ± 10	20 ± 15	18 ± 11	8 ± 7	5 ± 7
*P. alpina*	42 ± 9	25 ± 9	10 ± 7	13 ± 9	9 ± 6	1 ± 3

The PLS-DA with full CV classification of the pollen spectra reconstructed by the NMF approach results with higher success rate (82%) compared to PLS-DA results of the previous preprocessing procedures (compare Table 4 with Tables 1 and 2). The success rates for *Bromus inermis* and *Lolium perenne* are slightly higher (65 and 71%, Table 4) compared to the classification results of approach 1 (46 and 64%, Table 2). Nevertheless, the PCA results (ESM Fig. S3) indicate that the variation within the NMF-decomposed spectra might still be slightly affected by bands from paraffin, as indicated by the variation in the CH_2_ deformation at 1460 cm^−1^ that on the one hand is assigned to lipids in the pollen [16], but that could also be present due to residual paraffin contributions (ESM Fig. S3, right column, loadings of PC 2 and PC 6).

**Table 4:** Results of PLS-DA classification of spectra from paraffin-embedded pollen reconstructed from NMF components 1, 3, 4, and 5 (cf. Scheme 1, approach 2). See Table 3 and Figs. 3 and 4 for details on NMF components and NMF reconstruction of spectra. Nine latent variables were used. The results are based on full cross-validation

Output class	Target class				
	*A. odoratum*	*B. inermis*	*H. bulbosum*	*L. perenne*	*P. alpina*
*A. odoratum*	185	2	1	15	12
*B. inermis*	1	130	9	14	3
*H. bulbosum*	2	42	192	8	0
*L. perenne*	6	17	1	140	4
*P. alpina*	4	8	6	19	181
Success rate (SR)	93%	65%	92%	71%	91%
Overall SR	82%				

### Correction of the spectra using EMSC with a paraffin constituent spectrum

**Fig. 5:**
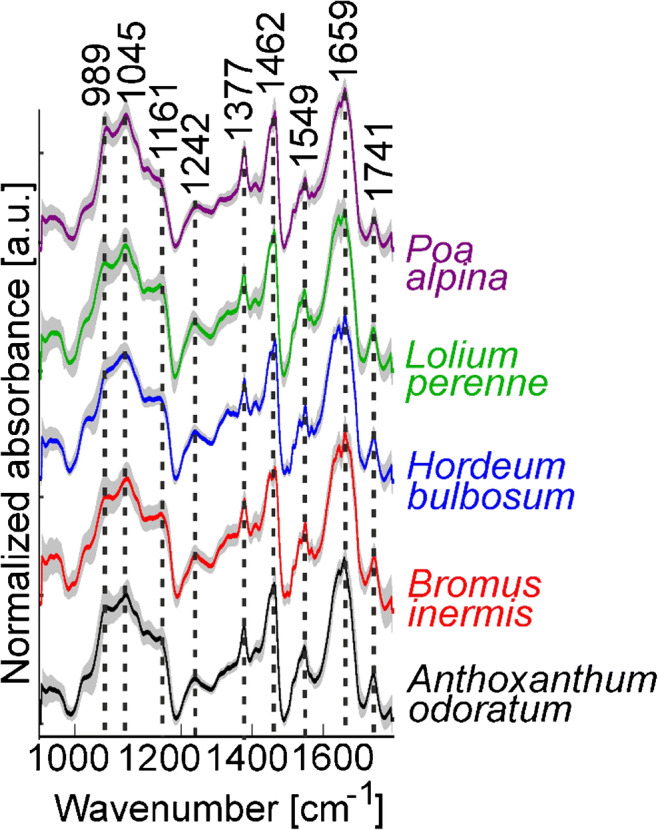
FTIR microspectra of paraffin-embedded pollen samples of the five grass species after correction using EMSC model with paraffin constituent spectrum (cf. Scheme 1, approach 3). Each spectrum is an average of 200 individual, corrected spectra (corresponding to 200 pollen grains). The standard deviation for each group of spectra is marked in gray

EMSC can be used to correct scattering and other non-absorption effects in FTIR data [35, 54, 55]. This is achieved by executing the model-based normalization with the help of a reference spectrum. In the preprocessing for approach 1 (cf. Scheme 1 and “Selection of non-affected spectral ranges” section), we used a simple EMSC model with linear and quadratic terms and the mean spectrum of the spectral data set [35]. Here, in approach 3 (cf. Scheme 1), we used the complex EMSC with a modeled paraffin contribution. We assume that the spectra are composed of two components, a paraffin and a pollen constituent. In order to apply EMSC on the data set, the pollen constituent spectrum was chosen as a reference spectrum, and an averaged pure paraffin spectrum was added into the algorithm as discussed previously [44]. As a result, the spectra are normalized so that particularly the bands at 1377 and 1462 cm^−1^ show less variation between the spectra from the five species (Fig. 5). For the classification, this would mean that the variation induced by the differences in the paraffin-embedding medium can be minimized and that classification is only based on the spectral contributions by the pollen grains themselves.

The PLS-DA with full CV classification of the pollen spectra preprocessed by the complex EMSC approach results with the highest success rate (83%) of all the tested approaches (Table 5). In particular, the success rate for *Bromus inermis* is higher (63% in Table 5) compared to the classification of the data set corrected using approach 1 (49% in Table 2). This indicates that the already very promising classification results obtained in the previous study on 11 plant species [31] can be improved even further by optimizing the spectral preprocessing step. In general, approach 2 (the NMF approach, Table 4) and approach 3 (the complex EMSC approach, Table 5) result with relatively similar success rates. For all preprocessing procedures, the success rates vary regarding the different pollen species. The pollen spectra of the species *Anthoxanthum odoratum*, *Hordeum bulbosum*, and *Poa alpina* can be classified well (Table 5, > 90%), while the identification of the spectra belonging to *Bromus inermis* and *Lolium perenne* is more challenging, with success rates of 63 and 69%, respectively.

**Table 5:** Results of PLS-DA classification of spectra from paraffin-embedded pollen corrected using EMSC model with paraffin constituent spectrum (cf. Scheme 1, approach 3). Eleven latent variables were used. The results are based on full cross-validation

Output class	Target class				
	*A. odoratum*	*B. inermis*	*H. bulbosum*	*L. perenne*	*P. alpina*
*A. odoratum*	187	3	1	16	10
*B. inermis*	1	126	7	14	2
*H. bulbosum*	2	44	197	7	0
*L. perenne*	5	19	0	136	4
*P. alpina*	4	8	4	23	184
Success rate (SR)	94%	63%	94%	69%	92%
Overall SR	83%				

### Classification by hierarchical cluster analysis and principal component analysis

The success rates for the full cross-validation PLS-DA-based classification indicate that the three approaches of minimizing the paraffin contribution to the spectra, namely (i) omitting a part of the spectrum (approach 1), (ii) non-negative matrix factorization (approach 2), and (iii) normalization of the paraffin signals by EMSC (approach 3), lead to a similar ability to discriminate the pollen spectra from the species *Anthoxanthum odoratum*, *Hordeum bulbosum*, and *Poa alpina*, and a less efficient classification of the two species *Bromus inermis* and *Lolium perenne* within the data set*.* It has been shown before that the spectra of some grass pollen species have more unique spectral features than others, so that their discrimination within a data set of similar pollen species is less difficult [20, 23]. In order to assess intra- versus interspecies differences, a hierarchical cluster analysis was performed, using the spectral data obtained by approach 3 (Scheme 1, left blue box). This pretreatment has the advantage that no supervision is needed, and automated pattern recognition tools could be developed for a fast identification of the spectra.

**Fig. 6:**
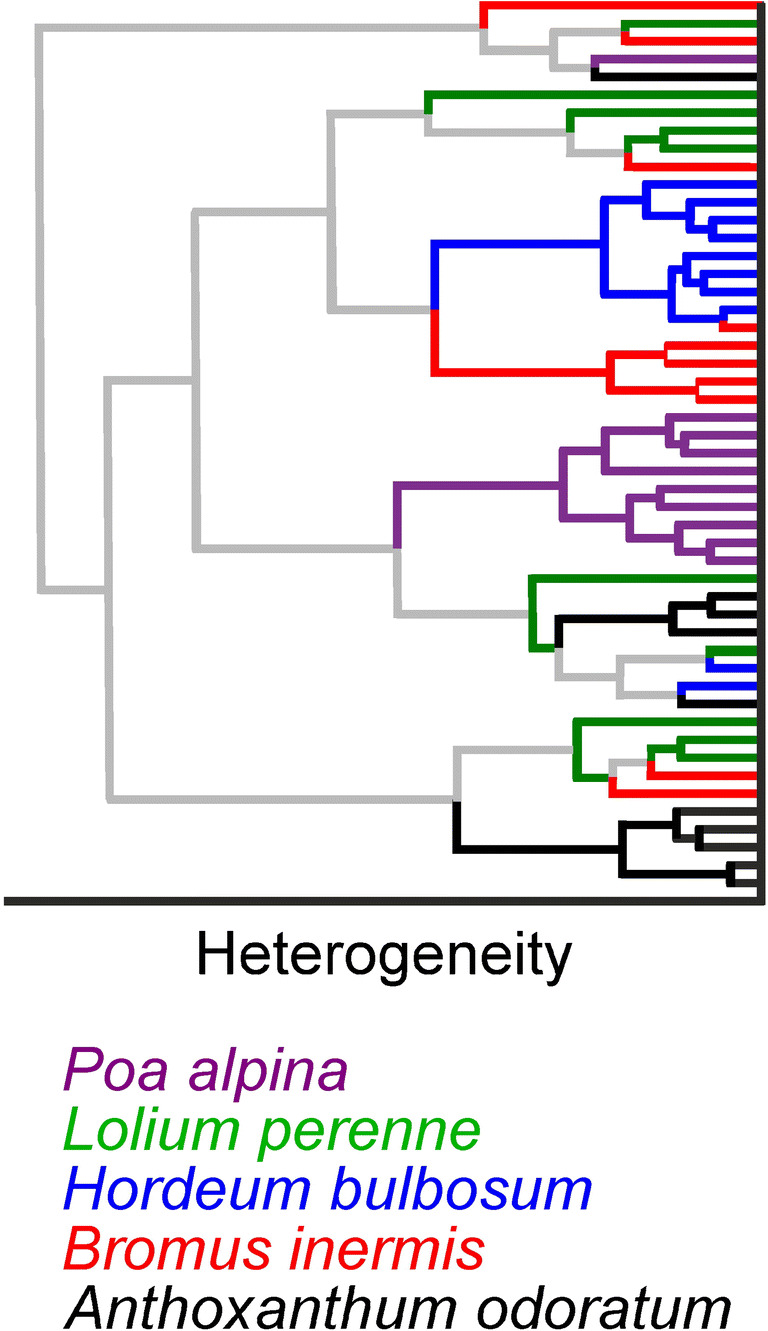
Dendrogram obtained after hierarchical cluster analysis (HCA) with 50 pollen spectra from the five indicated grass species, using the full spectral range from 800 to 1800 cm^−1^. Each spectrum in the analysis is an average of the ~ 20 pollen grain spectra of one individual plant (cf. Scheme 1, approach 3). HCA was executed using Euclidean distances and Ward’s algorithm. The colored branches correspond to the font color with which the respective pollen species is listed

The hierarchical cluster analysis was carried out with the average spectra of 20 single pollen spectra of each sample, leading to 50 spectra in total, using Euclidean distances and Ward’s algorithm*.* The resulting dendrogram is shown in Fig. 6. Most of the spectra of *Poa alpina* (Fig. 6, purple branches), *Anthoxanthum odoratum* (Fig. 6, black branches), and *Hordeum bulbosum* (Fig. 6, blue branches) are clustered almost exclusively in respective groups. This is in good agreement with the PLS-DA identification discussed above and indicates low variances within the spectra of the respective species. The high similarity of the majority of spectra from *Bromus inermis* (Fig. 6, red branches) to those of *Hordeum bulbosum* (Fig. 6, blue branches) agrees with the high number of *Bromus inermis* spectra that are misclassified in the PLS-DA as *Hordeum bulbosum* spectra (cf. Table 5). We therefore conclude on a high similarity of the composition of the pollen from these two species, in agreement with the close relationship of the tribes Hordeeae (Triticeae) and Bromeae within the Pooideae subfamily [56, 57]. The cluster in the dendrogram that comprises all except one spectrum from *Poa alpina* (Fig. 6, purple branches) is very similar to a group of spectra that contains average pollen spectra from *Anthoxanthum odoratum* and *Lolium perenne* plants (Fig. 6, black branches and green branches, respectively), also in agreement with the misclassification by PLS-DA of several individual spectra from *Anthoxanthum odoratum* as *Lolium perenne* and *Poa alpina*, and vice versa (Table 5). Moreover, it can be concluded that the chemical composition of these pollen has more similarities compared to those from the other species, in agreement with the fact that all of them belong to the Poeae/Aveneae tribe complex [56].

**Fig. 7:**
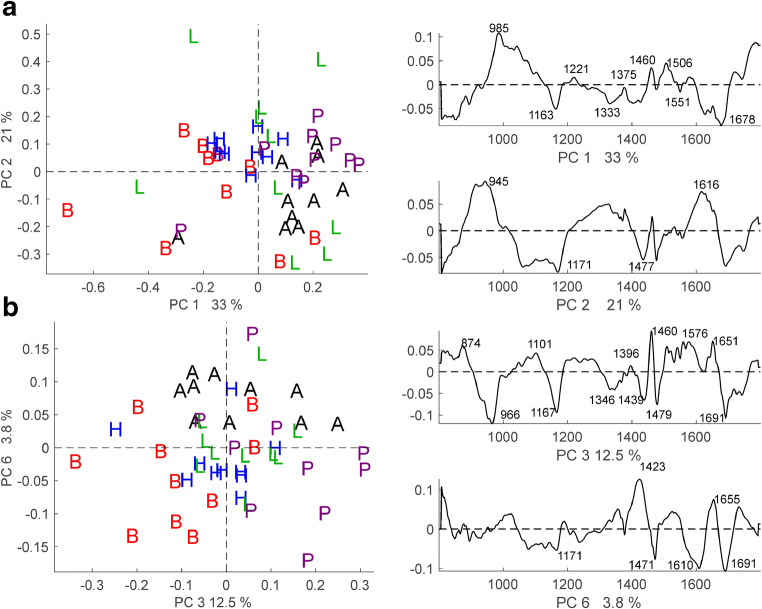
Principal component analysis (PCA) of 50 pollen spectra from the five indicated grass species, using the full spectral range from 800 to 1800 cm^−1^. Each spectrum in the analysis is an average of the ~ 20 pollen grain spectra of one individual plant corrected using EMSC with a paraffin constituent (cf. Scheme 1, approach 3). **a** Scores plot and corresponding loadings of PC 1 and PC 2. **b** Scores plot and corresponding loadings of PC 3 and PC 6. Each color representing the respective pollen species. Abbreviations: A, *Anthoxanthum odoratum* (black symbols); B, *Bromus inermis* (red symbols); H, *Hordeum bulbosum* (blue symbols); L, *Lolium perenne* (green symbols); P, *Poa alpina* (purple symbols)

In a PCA, the differences between the spectra of the five pollen species can be identified based on the loadings spectra. Figure 7 a shows the scores plot and corresponding loadings of the first and second principal components of a PCA with the average spectra of the 50 plants. The first and second PCs explain 54% of the total variance in the data set. As visible in the scores plot in Fig. 7 a, the mostly positive score values regarding the first PC of spectra from *Poa alpina* and *Anthoxanthum odoratum*, as well as most spectra from *Lolium perenne*, confirm the high similarity of the pollen composition in these two species. Similarly, the spectra from *Bromus inermis* and *Hordeum bulbosum* show mostly negative score values regarding the first PC (Fig. 7a). According to the loadings in Fig. 7 a, the most pronounced differences between the spectra from the *Bromus inermis*/*Hordeum bulbosum* group and those from the two species *Poa alpina* and *Anthoxanthum odoratum* are in the broad bands around 945 cm^−1^ (second PC) and 1678 cm^−1^ (first PC) that could be assigned to molecular vibrations of carbohydrates and proteins, respectively [14, 49, 53]. This would lead to the conclusion that pollen from *Bromus inermis* can be discriminated from *Poa alpina* and *Anthoxanthum odoratum* based on a different carbohydrate and protein composition. The scores plot in Fig. 7 b shows that separation of *Poa alpina* and the *Bromus inermis*/*Hordeum bulbosum* group from the other species is also possible based on the variance in the third PC. According to the corresponding loading spectra in Fig. 7 b, the discrimination is caused by signals that can be assigned to carbohydrates at 966 cm^−1^, to sporopollenin at 1167 and 1610 cm^−1^, here tentatively assigned to lipids at 1423 and 1460 cm^−1^, and to proteins at 1651 and 1691 cm^−1^ [16, 18].

### Pattern recognition for classification of grass pollen spectra from independent populations

The PLS-DA models discussed in the previous sections (“Selection of non-affected spectral ranges” and “Correction of the spectra using EMSC with a paraffin constituent spectrum”) show high success rates for the discrimination of the three pollen species *Anthoxanthum odoratum*, *Hordeum bulbosum*, and *Poa alpina* in a leave-one-out approach, where each individual spectrum of each sample is tested separately. Nevertheless, in such a setting, the model is trained with spectra from different plants, but from the same population as those of the pollen that is identified. A robust, reliable discrimination method should include the possibility to identify pollen spectra that come from different plant populations as well. In our experiments, the plants in each of the five species originate from two different populations. Therefore, a PLS-DA model was constructed using spectra from just one population per species, amounting to 502 spectra. The success rates were obtained by using the respective other population from each species as an independent test set, comprising other 502 spectra. The results for the identification of the unknown populations by PLS-DA are shown in the upper section of Table 6. Comparing the success rates with the results obtained in the leave-one-spectrum-out approach above (Table 4), we find that the success rates are only slightly lower for the species *Anthoxanthum odoratum*, *Hordeum bulbosum*, and *Poa alpina* when spectra come from an unknown population. Nevertheless, they are very low in those species, where success rates werealready low in the leave-one-spectrum-out identification, that is, in *Bromus inermis* and *Lolium perenne* (compare the upper section of Table 6 with Table 5), with the success rate for classification of the former drops from 63% to 26%. We assign this low success rate to a greater variation between the different populations in these two species. Similar observations have been reported for other grass species with bulk FTIR and MALDI mass spectrometry approaches as well and have been discussed in the greater context of adaptive variation [23, 58]. We have also used the second derivatives of the spectra, which yielded similar success rates (ESM Table S1).

**Table 6:** Results of the classification of the spectra of paraffin-embedded pollen corrected using an EMSC model with paraffin constituent spectrum (cf. Scheme 1, approach 3). Three classifiers were used for the classification: partial least square discriminant analysis using nine latent variables, artificial neural networks, and random forest. Training of the classification models was based on spectra from only one population for each grass species, while the independent validations were conducted using the other respective population for each species

Output class	Target class				
	*A. odoratum*	*B. inermis*	*H. bulbosum*	*L. perenne*	*P. alpina*
Partial least square discriminant analysis*					
*A. odoratum*	82	4	0	12	1
*B. inermis*	0	26	6	4	4
*H. bulbosum*	8	27	94	7	2
*L. perenne*	4	36	1	46	4
*P. alpina*	6	7	3	31	87
Success rate (SR)	82%	26%	90%	46%	89%
Overall SR	67%				
Artificial neural network					
*A. odoratum*	80	1	0	4	1
*B. inermis*	0	30	20	4	5
*H. bulbosum*	10	22	81	2	1
*L. perenne*	6	39	0	66	2
*P. alpina*	4	8	3	24	89
Success rate (SR)	80%	30%	77%	66%	91%
Overall SR	69%				
Random forest					
*A. odoratum*	75	31	2	23	6
*B. inermis*	1	26	10	4	15
*H. bulbosum*	6	4	87	1	3
*L. perenne*	10	34	2	47	2
*P. alpina*	8	5	3	25	72
Success rate (SR)	75%	26%	84%	47%	73%
Overall SR	61%				

*Table S1 (see ESM) shows the results for the classification of 2nd derivative spectra

Apart from the linear classifier PLS-DA, we used machine learning for the identification of spectra from the respective unknown populations. A feed-forward ANN was trained with the same set of 502 spectra, divided into a training, validation, and internal test set, and tested with 502 spectra from the other respective populations. The success rates were very similar, with a higher number of correct species assignment in *Lolium perenne* and similar misclassification, e.g., assignment of *Lolium perenne* as *Poa alpina* (Table 5, middle section). The slightly diminished success rate for the identification of *Hordeum bulbosum* compared to the PLS-DA classifier is balanced, considering a 66% correct identification of the spectra from *Lolium perenne* pollen that is an improvement compared to the PLS-DA model (compare top and middle sections in Table 6). Consistent with the results of HCA (Fig. 6) and PCA (Fig. 7), almost all incorrectly assigned spectra of *Hordeum bulbosum* are labeled with *Bromus inermis* as output class, also in agreement with the close phylogenetic relationship of the two species mentioned above [56, 57]. Identification using a random forest algorithm as another machine learning approach results in similar success rates as the PLS-DA model in the case of *Bromus inermis* and *Lolium perenne*, but lower numbers of correct identification than PLS-DA and ANN for the other three species (Table 5, last section). Changing the number of trees in the RF from 300 (cf. results in Table 3), determined to be optimum, to higher numbers, results in similar success rates.

The strong decrease in classification success in *Bromus inermis* when an unknown population must be identified (compare the respective columns in Table 4 and in the three sections of Table 5) and the quite high success rates for other species are in agreement with the different intraspecies variance that was observed between populations of other Poaceae species [23, 58]. Especially in *Anthoxanthum odoratum* and also *Poa alpina* that show highest success rates here (Table 5), the ability to distinguish spectra from different populations of the same species was challenging based on FTIR spectra [23] but could be achieved using other chemical information of the pollen samples [58].

The fact that identification is based here on spectra from individual pollen grains rather than averages from one plant adds another source of variation here, as was recently also discussed when we compared different spectroscopic methods that probe either bulk samples or individual pollen grains and their potential for pollen identification [24]. Nevertheless, the possibility to study pollen spectra in mixtures could in the future open possibilities for FTIR imaging-based identification of mixed grass pollen samples, similar to existing high-throughput and mapping approaches [9, 10].

## Conclusions

The results indicate that different spectral preprocessing strategies to minimize the influence of unwanted paraffin spectral contributions in the FTIR microspectra of individual grass pollen grains are feasible. These spectral preprocessing procedures lead to meaningful classification results of pollen samples from the five very similar Pooideae grass species *Anthoxanthum odoratum*, *Bromus inermis*, *Hordeum bulbosum*, *Lolium perenne*, and *Poa alpina*. The analysis shows that, while classification of the spectra is possible with good success rates in spite of strong paraffin absorption, the elimination of the paraffin spectral features is desirable, since the effect by the paraffin embedding depends on the pollen grain morphology and has a strong influence on the classification. The relative amount of the paraffin contribution was characterized by NMF of the spectra and by the complex EMSC model with paraffin constituent. Both NMF and complex EMSC approaches improve the classification success rates, compared to a removal of the spectral region that contains the strongest absorption bands of paraffin.

Following the EMSC-based correction approach using a paraffin constituent spectrum, requiring a decision by the operator, it is possible to identify spectra from different populations applying PLS-DA, as well as ANN and RF machine learning. This suggests that both the spectral preprocessing and the identification of the spectra can in principle be included in an automated analysis of pollen samples, e.g., as collected from typical pollen traps.

Using average spectra from all pollen samples of each of the 50 individual plants, the spectral variation within and between species, together with the particular mis/-classification results for the investigated species, is in agreement with the systematics within the Poaceae family. Furthermore, success rates for classification of unknown populations reveal a variation of chemical differences between respective different populations for the five species. Therefore, in future work, FTIR microspectroscopy will be combined with other microscopic analytical methods that give single pollen grain spatial resolution and sensitivity, namely Raman microscopy [11, 29] and MALDI imaging [9]. Such a multimodal single pollen probing will be a logical continuation of recent experiments combining the complementary chemical information of these methods [18, 24].

## Electronic supplementary material

ESM 1 (PDF 500 kb)
