# Supplementary material for: Discrimination of grass pollen of different species by FTIR spectroscopy of individual pollen grains
Source: Anal Bioanal Chem. 2020 Apr 29;412(24):6459–74. doi: 10.1007/s00216-020-02628-2 (PMC7442581; doi:10.1007/s00216-020-02628-2)
Supplement: Supplementary file 1 — (PDF 500 kb) [file 216_2020_2628_MOESM1_ESM.pdf]

Supplementary Material

Discrimination of grass pollen of different species by FTIR spectroscopy of individual pollen grains

Sabrina Diehn[1], *Boris Zimmermann*[2], *Valeria Tafintseva*[2], *Murat Bağcıoğlu*[2], *Achim Kohler*[2], *Mikael Ohlson*[3], *Siri Fjellheim*[4] *and Janina Kneipp*[1*]

[1] Department of Chemistry, Humboldt-Universität zu Berlin, Brook-Taylor-Straße 2, 12489 Berlin, Germany

[2] Faculty of Science and Technology, Norwegian University of Life Sciences, 1432 Ås, Norway

[3] Faculty of Environmental Sciences and Natural Resource Management, Norwegian University of Life Sciences, 1432 Ås, Norway

[4] Faculty of Biosciences, Norwegian University of Life Sciences, 1432 Ås, Norway

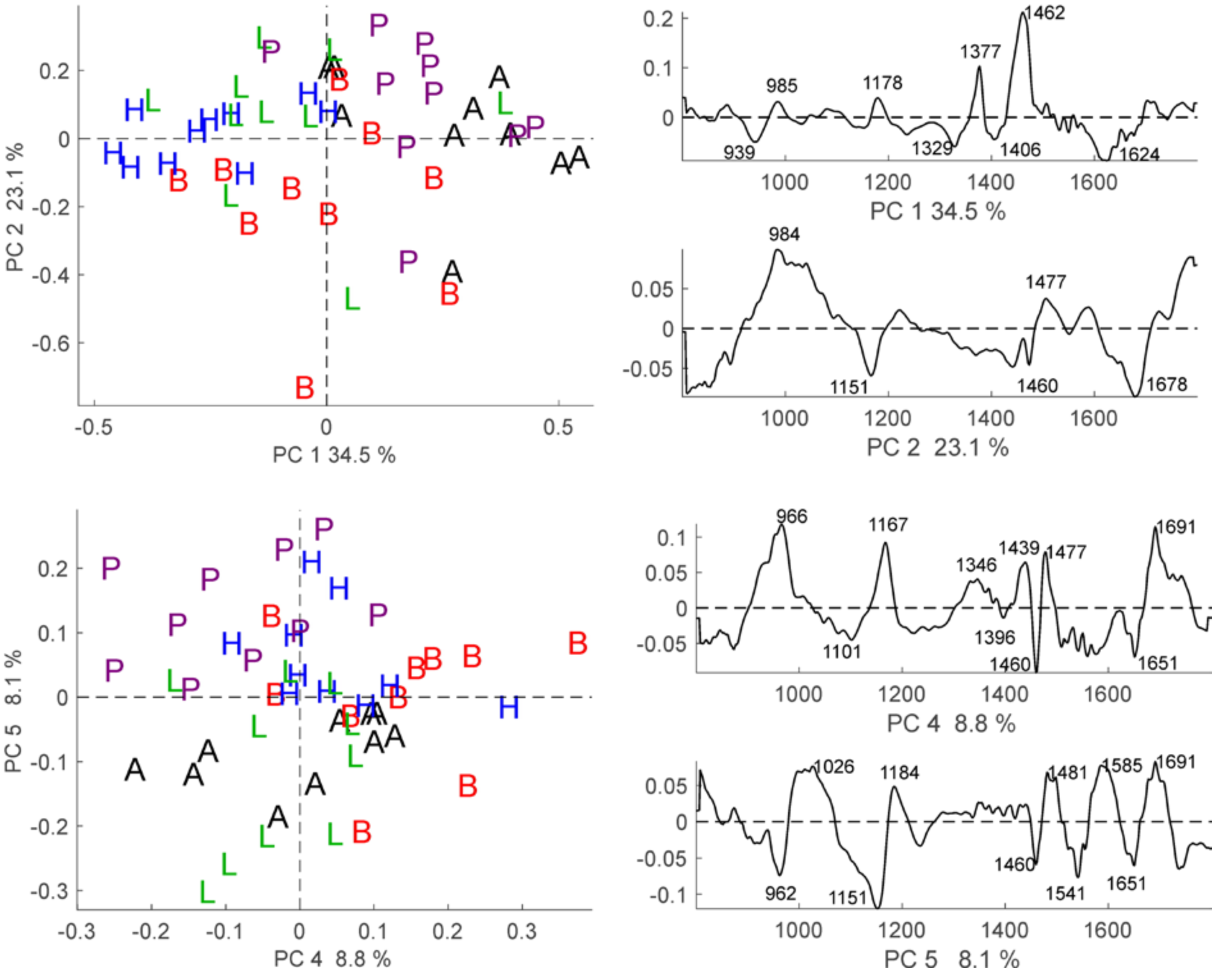

Figure S1: Scores and loadings from a principal component analysis (PCA) with 50 pollen spectra from the five indicated grass species using the full spectral range from 800 – 1800 cm^{-1}. Each spectrum in the analysis is an average of the ~20 pollen grain spectra of one individual plant. Spectra were baseline-corrected and normalized, no correction for the contribution by embedding paraffin was undertaken. **(A)** Scores plot and corresponding loadings of PC 1 and PC 2. **(B)** Scores plot and corresponding loadings of PC 4 and PC 5. Each color and symbol represents the respective grass species. A, *Anthoxanthum odoratum* (black symbols) B, *Bromus inermis* (red symbols) H, *Hordeum bulbosum* (blue symbols) L, *Lolium perenne* (green symbols) P, *Poa alpina* (purple symbols).

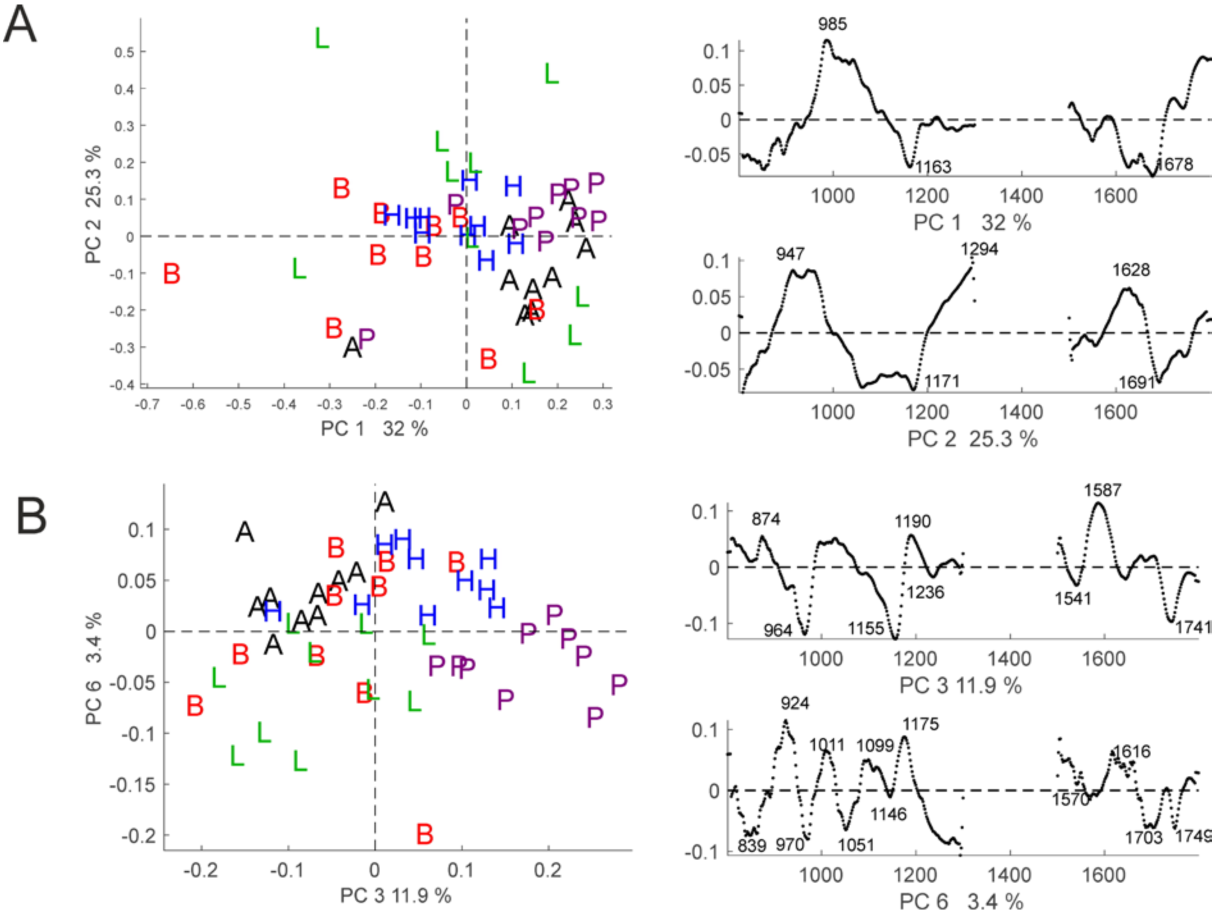

Figure S2: Scores and loadings from a principal component analysis (PCA) with 50 pollen spectra from the five indicated grass species omitting the spectral range from 1300 – 1500 cm^{-1} that contains strong contributions by paraffin vibrational modes (cf. Scheme 1 in the manuscript, approach 1). Each spectrum in the analysis is an average of the ~20 pollen grain spectra of one individual plant. **(A)** Scores plot and corresponding loadings of PC 1 and PC 2. **(B)** Scores plot and corresponding loadings of PC 3 and PC 6. Each color and symbol represents the respective grass species. A, *Anthoxanthum odoratum* (black symbols) B, *Bromus inermis* (red symbols) H, *Hordeum bulbosum* (blue symbols) L, *Lolium perenne* (green symbols) P, *Poa alpina* (purple symbols). To better indicate the region that was excised from the spectra, the loadings are shown as individual data points rather than as line plots.

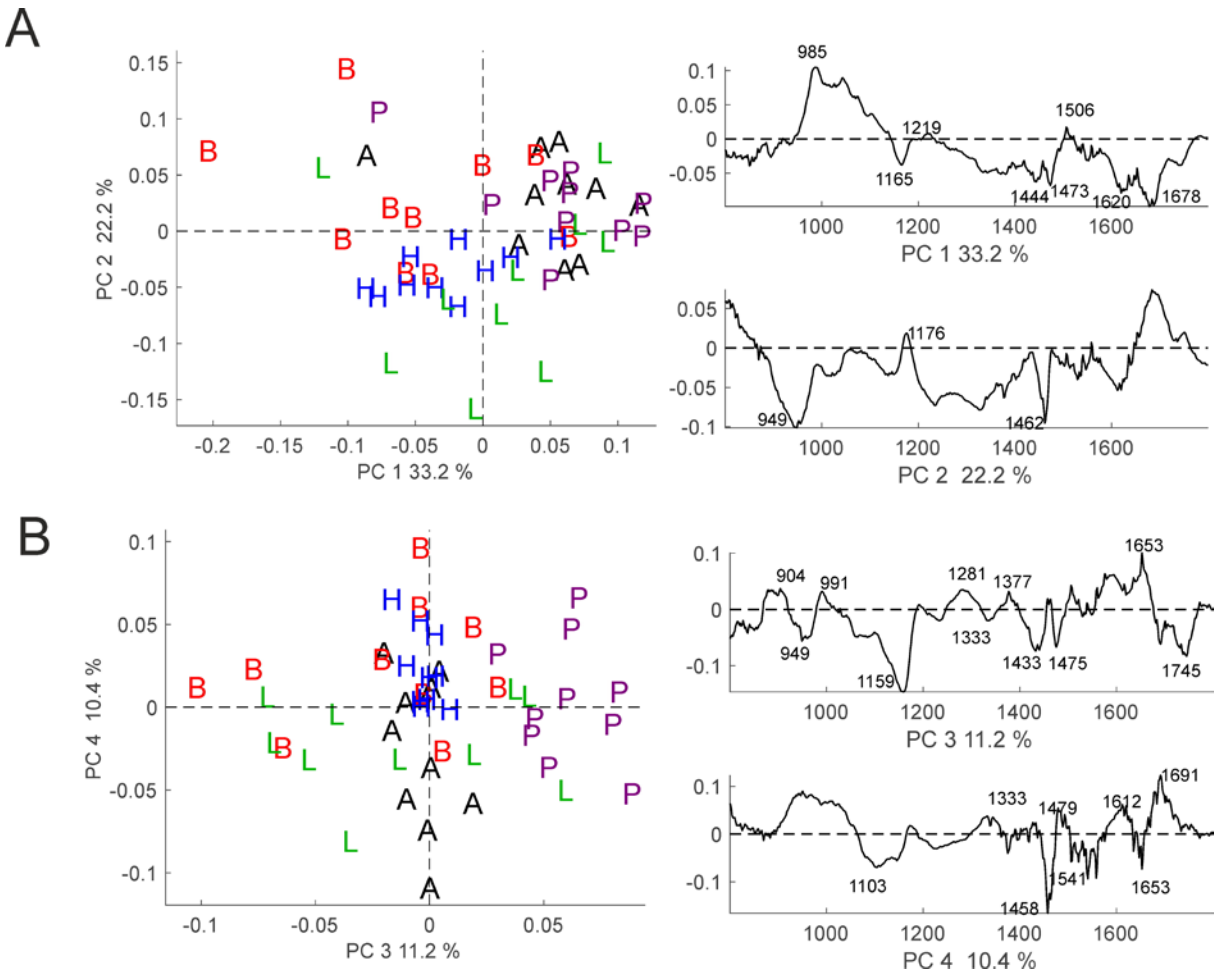

Figure S3: Scores and loadings from a principal component analysis (PCA) with 50 pollen spectra from the five indicated grass species using the full spectral range from 800 – 1800 cm^{-1}. Each spectrum in the analysis is an average of the ~20 pollen grain spectra of one individual plant calculated after reconstruction following a non-negative matrix factorization (NMF) (cf. Scheme 1 in the manuscript, approach 2). After pre-processing, the spectra were decomposed by NMF using six components (cf. Table 3) and reconstructed without the two components that represent the spectral contribution by paraffin. **(A)** Scores plot and corresponding loadings of PC 1 and PC 2. **(B)** Scores plot and corresponding loadings of PC 3 and PC 4. Each color and symbol represents the respective grass species. A, *Anthoxanthum odoratum* (black symbols) B, *Bromus inermis* (red symbols) H, *Hordeum bulbosum* (blue symbols) L, *Lolium perenne* (green symbols) P, *Poa alpina* (purple symbols).

Table S1: Results of the PLS-DA classification of the spectra of paraffin-embedded pollen corrected using an EMSC model with paraffin constituent spectrum (cf. Scheme 1, approach 3) and partial least square discriminant analysis using 9 latent variables. 2nd derivatives were taken using the Savitzky-Golay algorithm with a window size of 31 points. Training of the models was based on spectra from only one population for each grass species, while the independent validations were conducted using the other respective population for each species.

**Target class**	*A. odoratum*	*B. inermis*	*H. bulbosum*	*L. perenne*	*P. alpina*
**Output class**					
**2nd derivative**					
A. odoratum	88	0	1	18	0
B. inermis	0	40	21	6	7
H. bulbosum	0	21	74	4	2
L. perenne	4	34	1	52	0
P. alpina	8	5	7	20	89
Success rate	88 %	40 %	71 %	52 %	91 %
